# An Eleven-Year Retrospective Study of Endogenous Bacterial Endophthalmitis

**DOI:** 10.1155/2015/261310

**Published:** 2015-01-31

**Authors:** Takashi Nishida, Kyoko Ishida, Yoshiaki Niwa, Hideaki Kawakami, Kiyofumi Mochizuki, Kiyofumi Ohkusu

**Affiliations:** ^1^Department of Ophthalmology, Gifu University Graduate School of Medicine, Gifu, Japan; ^2^Department of Ophthalmology, Toho University Ohashi Medical Center, 2-17-6 Ohashi, Meguro-ku, Tokyo 153-8515, Japan; ^3^Department of Ophthalmology, Gifu Prefectural General Medical Center, Gifu, Japan; ^4^Department of Ophthalmology, Gifu Municipal Hospital, Gifu, Japan; ^5^Department of Microbiology, Tokyo Medical University Graduate School of Medicine, Tokyo, Japan

## Abstract

*Purpose*. To determine the clinical features, microbial profiles, treatment outcomes, and prognostic factors for endogenous bacterial endophthalmitis (EBE). *Methods*. The medical records of 27 eyes of 21 patients diagnosed with EBE for 11 years were reviewed. Collected data included age, site of infection, visual acuities (VAs), microbial profiles, and treatment regimen. *Results*. The mean age was 68.5 years. Gram-positive organisms accounted for 76.2%, while gram-negative ones accounted for 19.0%. *Staphylococcus aureus* was the most common causative organism (52.3%) of which 72.7% was *methicillin-resistant S. aureus*. A final VA of ≥20/40 was achieved in 44% and 20/200 or better was in 64%. Eyes with initial VA of ≥20/200 (*P* = 0.003) and focal involvements (*P* = 0.011) had significantly better final VA. Initial VA (*P* = 0.001) and the interval between onset of ocular symptoms and intravitreal antibiotic injection (*P* = 0.097) were associated with final VA in eyes receiving intravitreal antibiotics. *Conclusions*. EBE is generally associated with poor visual outcome; however the prognosis may depend on initial VA, extent of ocular involvement, and an interval between onset of ocular symptoms and intravitreal antibiotic injection. Early diagnosis and early intravitreal injection supplement to systemic antibiotics might lead to a relatively good visual outcome.

## 1. Introduction

Endogenous bacterial endophthalmitis (EBE) is rare and accounts for 2% to 8% of all cases of endophthalmitis [1–3]. It is a damaging disease, and the visual prognosis is generally poor with more than one-half of eyes becoming blind despite treatment [1–4]. The systemic pathological conditions predisposing an eye to endophthalmitis include malignancies, alcoholism, cardiovascular diseases, diabetes mellitus, indwelling catheters, bone and joint diseases, intravenous drug use, hemodialysis, trauma, and cirrhosis of the liver [1–3, 5, 6]. Endogenous bacterial endophthalmitis is both an ocular and extraocular disease [1, 3].

The visual outcome of endogenous endophthalmitis is poorer than that of exogenous endophthalmitis. The main factors associated with the poor prognosis include the virulence of the causative organisms, compromised host conditions, and a delay in diagnosis and treatment [1–3, 6]. EBE can be an unusual complication of systemic bacterial infections with severe consequences if left untreated. Patients at high risk for bacteremia who present with ocular complaints should have a thorough funduscopic examination to rule out endophthalmitis [6, 7].

There are no standardized diagnosis or treatment guidelines for endogenous bacterial endophthalmitis because of its low incidence, inadequate data on the treatment regimens, and the lack of long-term follow-up results [2, 3, 8].

The purpose of this study is to evaluate the clinical features, microbiological spectrum, treatment regimens, and outcomes of EBE in three centers in Japan during an 11-year period. We also aimed to assess associated factors for visual prognosis.

## 2. Patients and Methods

### 2.1. Patients

We reviewed the medical records of consecutive patients diagnosed with EBE between January 2002 and June 2013. All of the patients were referred to three hospitals of Gifu prefecture: Gifu University Graduate School of Medicine, Gifu Municipal Hospital, and Gifu Prefectural General Medical Center. The patients were diagnosed with EBE by constitutional symptoms, decrease in visual acuity (VA), ocular pain, hypopyon, subretinal lesions, and anterior and/or posterior uveitis. The uveitis was confirmed by cultures of the blood, vitreous, or aqueous humor in all cases. The findings in 3 of these patients (Patients 6, 7, and 12) were reported previously [9–11].

Patients were excluded if they had a history of ocular trauma or ocular surgery within 1 year of the onset of the infection or evidence of a primary external ocular infection such as infectious keratitis or filtering bleb infection [2, 12].

The medical records were reviewed. Collected date included the age, sex, presenting complaints, underlying systemic infections, preexisting medical conditions, source of infection, laterality, VA, microbial profiles, treatment methods, and initial and final VAs. The main outcome measure was the best-corrected VA at the final follow-up examination. In earlier reports [9, 12], a best-corrected VA of counting fingers (CF) or better was classified as being a good visual outcome for the statistical analyses; however in this report we used 20/200 or better as a good visual outcome. Other outcome measures included the results of microbiological investigations and anatomical and clinical outcomes.

### 2.2. Statistical Analysis

Fisher's exact test, Chi-square test, Kruskal-Wallis test, Mann-Whitney *U* test, and Spearman's rank correlation coefficient were used to determine the significance of any differences. Logistic regression and multiple regression analysis were also used to detect associated factors for visual prognosis. The odds ratio was calculated, and the 95th percentile confidence intervals (95% CI) were determined. The decimal VA values were converted to the logarithm of the minimum angle of resolution (logMAR) units. For a visual acuity less than CF the following arbitrary logMAR values were used: CF = 2.00 logMAR units, hand motion = 2.30 logMAR units, light perception = 2.60 logMAR units, and no light perception = 2.90 logMAR units [13, 14].

A *P* value of <0.05 was considered to be significant. All statistical analyses were performed using SPSS software version 16.0 (SPSS Japan, Tokyo, Japan).

## 3. Results

### 3.1. Patient Characteristics and Systemic Features

The study was approved by the institutional review boards. Twenty-seven eyes of 21 Japanese patients were included in the current study. Patient characteristics and systemic features are shown in Table 1. The mean age at presentation was 68.5 ± 9.7 years (range, 41 to 80 years). The mean follow-up period was 76.3 weeks with a range of 2 to 520 weeks. There were 14 (66.7%) men and 7 (33.3%) women. Thirteen (61.9%) patients had community-acquired infections, and the others (38.1%) had hospital-acquired infections. The number of patients with EBE was 5 (23.8%) in the spring, 3 (14.3%) in the summer, 8 (38.1%) in the autumn, and 5 (23.8%) in the winter.

All patients had one or more preexisting medical conditions that predisposed to the development of EBE. The most common medical condition was diabetes mellitus (13 patients, 61.9%), followed by hypertension (*n* = 6, 28.6%), gastrointestinal disorders (*n* = 5, 28.8%), cardiac disease (*n* = 5, 28.8%), malignancy (*n* = 5, 28.8%; 4 solid organ malignancies and 1 hematologic malignancy), urological diseases (*n* = 5, 28.8%), and hemodialysis (*n* = 2, 9.5%) (Table 1). Systemic steroids were being used to treat an underlying illness in 5 (28.8%) patients.

Extraocular infectious foci were identified in 17 (81.0%) patients, infective endocarditis was identified in 3 (14.3%) patients, pneumonia was identified in 2 (9.5%), soft tissue (skin and wound) infection was identified in 2 (9.5%), peritonitis was identified in 2 (9.5%), catheter-related infection was identified in 2 (9.5%), recent trauma (head and kidney) was identified in 2 (9.5%), liver abscess was identified in 1, urinary tract infection was identified in 1, burn was identified in 1, and psoas abscess was identified in one. The source of infection could not be identified in 4 patients.

Eighteen patients (85.7%) had an onset of systemic or ocular symptoms within 7 days prior to their admission, and 2 patients developed symptoms ≥4 weeks before admission (mean 6.0 days; range, <1 to 29 days). The most common initial systemic symptom was fever including cold-like symptoms in 16 (76.2%) patients, followed by diarrhea and vomiting (*n* = 1), anterior chest pain (*n* = 1), back pain (*n* = 1), myalgia (*n* = 1), and injection site abscess (*n* = 1).

At the first visit to a doctor, a high body temperature (>38°C) was noted in 16 (76.2%), elevated C-reactive protein (range, 3.69–30.23 mg/dL) in 16 (76.2%), and a high white blood cell count (12,630–24,560/*μ*L) in 11 (52.3%) of the 21 patients. The plasma level of *β*-D-glucan was measured in 14 patients and the range was 11.9–230 pg/mL, and it was considered positive in 1 patient when the cutoff value was set at 20 pg/mL [15].

### 3.2. Ocular Features

Five (23.8%) presented with right eye involvement, 10 (47.6%) with left eye involvement, and 6 (28.6%) with bilateral involvement (Table 2). The first ocular symptom was decreased vision (14 patients, 66.7%), floaters (5 patients, 23.8%), pain (2 patients, 9.5%), and eyelid swelling (1 patient, 4.8%). However, only 5 (23.8%) of 21 patients initially consulted with an ophthalmologist in a private office or hospital, and 16 (76.2%) sought help from other clinical departments, for example, internal medicine (*n* = 10), orthopedics (*n* = 2), emergency (*n* = 2), and surgery (*n* = 2). The mean interval between the onset of ocular signs or symptoms and the first visit to an ophthalmologist was 8.4 days (range, 1 to 30 days). A correct initial ocular diagnosis of bacterial endophthalmitis was made by ophthalmologist in 15 (71.4%) patients. Other initial ocular diagnoses were uveitis (*n* = 4, 19.0%), fungal endophthalmitis (*n* = 2, 9.5%), choroidal tumor (*n* = 1), and glaucoma (*n* = 1).

The principal sites of EBE [6] for the 27 eyes were diffuse posterior endophthalmitis in 10 (37.0%) eyes, posterior focal endophthalmitis in 13 (48.1%) eyes, and panophthalmitis in 4 (14.8%) eyes (Table 2).

### 3.3. Microbiology

The organism causing the endophthalmitis was identified by a positive culture from at least one body fluid source in 19 (90.5%) of the 21 patients (Table 2). In two of 21 patients, the pathogenic organism was not determined. Fungus was not detected in any of the samples. The organisms isolated from the blood, intraocular, and other cultures are shown in Table 3. Twenty-two organisms were identified in total. The blood was the highest source of a positive culture and 17 organisms were detected from 15 of 20 patients. A positive vitreous culture was obtained in 6 of 10 patients and aqueous humor in 2 of 6 patients. Two patients had positive central venous catheter tip cultures and a positive soft tissue culture including psoas. Diagnostic vitrectomy was performed in 1 patient (Patient 6) at 49 days after the appearance of ocular symptoms or signs because of high plasma level of *β*-D-glucan.

Eighteen gram-positive organisms were identified in 16 (76.2%) patients and 4 gram-negative pathogens in 4 (19.0%) patients.* Staphylococcus* species represented the most common group, and they were found in 12 (57.1%) patients, followed by* Streptococcus* species in 5 (23.8%) patients.

Among 12* Staphylococcus aureus *species, 8 cases were methicillin-resistant* S. aureus *(MRSA) and 3 were methicillin-sensitive* S. aureus* (MSSA). The primary site of* S. aureus* was at a trauma site including burns in 3 (2 MRSA and 1 MSSA), a soft tissue infection in 2 (2 MRSA), catheter-related infection in 2 (MRSA and MSSA), psoas abscess (MSSA) in 1, pulmonary abscess (MRSA) in 1, peritonitis (MRSA) in 1, and unknown foci (MRSA) in 1 (Table 2). All MRSA strains were sensitive to vancomycin, daptomycin, and linezolid. Three patients who had positive blood samples had mixed infections. One had a catheter-related infection due to MSSA and* S. agalactiae*, and the second had kidney trauma associated with MRSA and* Enterobacter cloacae*. The third had malignancy (postoperative stage of uterine cancer) associated with coagulase-negative* Staphylococcus* (CNS) and* S. agalactiae* which were the same organisms in the vitreous samples obtained during vitrectomy (Patient 3).

### 3.4. Treatment

The medications were started in an average of 8.1 days (range, <1 to 28 days) after the diagnosis of endophthalmitis. All (100%) of the 21 patients initially received intravenous antibiotics, usually *β*-lactam or carbapenem (Table 2). Seven (33.3%) of these cases were also given systemic antifungal agents concurrently. After the identification of causative organisms, the antifungal agents were discontinued and the systemic and/or intravitreal antibiotics were adjusted according to the species and sensitivity results. Fifteen (55.5%) of 27 eyes received intravitreal antibiotics: 1 mg of vancomycin in 14 eyes, 2 mg of ceftazidime in 11 eyes, and/or 500 *μ*g of meropenem in 1 eye. Four (14.8%) eyes had intravitreal antibiotics within 24 h of the diagnosis of endophthalmitis. Six (22.2%) eyes underwent initial therapeutic vitrectomy which was performed in an average of 16.8 days (range, 1–37 days) after the onset of the ocular symptoms or signs, and all were treated with intravitreal antibiotics during the vitrectomy. Intravitreal injections of steroids were not given to any eye. In 2 of the 6 eyes treated with vitrectomy, silicone oil tamponade was used. One of the 6 eyes (Patient 10, left eye) had subsequent vitrectomy and silicone oil tamponade with repeat intravitreal antibiotics for a retinal detachment 7 days after the first surgery but not during the active phase of the endophthalmitis.

### 3.5. Outcomes

**Figure 1:**
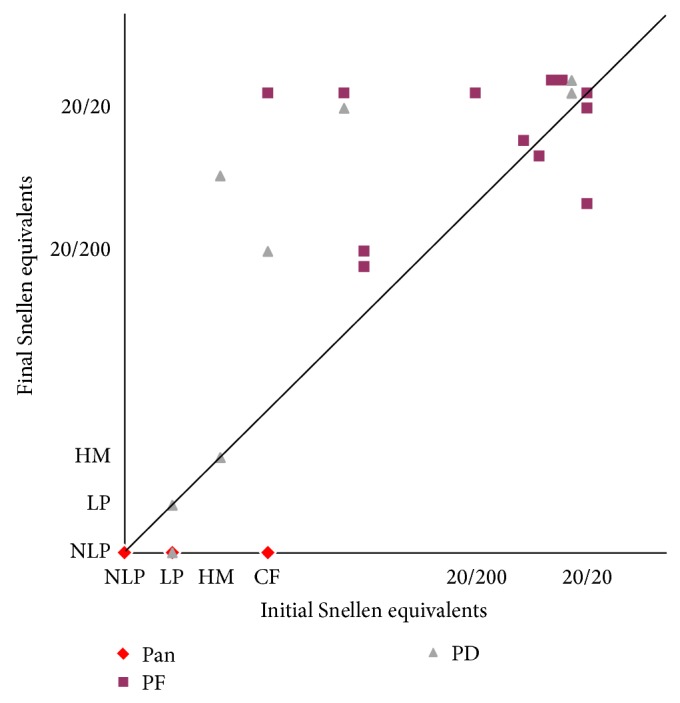
Visual outcome of endogenous bacterial endophthalmitis. CF, counting fingers; HM, hand motion; LP, light perception; NLP, no light perception (including enucleation); Pan, panophthalmitis; PD, posterior diffuse; PF, posterior focal.

The initial and final VAs for the 25 eyes of 20 patients for whom both data points were available (two eyes of one patient who could not tolerate the VA test because of critical general conditions) are shown in Figure 1 and each plot was classified with ocular involvement. An initial VA of 20/200 or better was found in 10 (40.0%) of 25 eyes and equal to or less than counting fingers in 11 (44.4%) eyes. A final VA of better than 20/200 was achieved in 16 (64.0%) and 20/40 or better in 11 (44.4%) of 25 eyes. Despite the treatments, the VA was <20/200 in 9 (36.0%) eyes at the latest follow-up examination and 3 eyes required enucleation and 2 had phthisis bulbi.

Univariate analyses were performed to identify the clinical factors associated with a final VA of ≥20/200 (Table 4). Eyes with an initial VA of ≥20/200 had a significantly better final visual outcome (Fisher's exact test: *P* = 0.003). Eyes with the focal type of endophthalmitis had a significantly better final VA (Chi-square test: *P* = 0.003). Diabetes mellitus was significantly associated with final VA of ≥20/200 (Fisher's exact test: *P* = 0.010). The sex, laterality, hypertension, systemic steroid treatments for underlying illness, infection place (community-acquired/hospital-acquired endophthalmitis), causative organs, and treatment with or without intravitreal antibiotics or vitrectomy were not significantly associated with final VA of ≥20/200 (Fisher's exact test: all *P* values > 0.05). To identify the associated factors for visual outcome, logistic regression analysis was used employing final VA of ≥20/200 as the outcome value and several parameters as explanatory variables. The latter included age, sex, laterality, initial visual acuity, extent of ocular involvement, presence or absence of diabetes mellitus or hypertension, infection place, causative organs, treatment with or without intravitreal antibiotics or vitrectomy, interval between onset of ocular symptom and ophthalmology consultation, and follow-up period. None was detected as an associated factor for final VA of ≥20/200 by logistic regression analysis.

Using the actual values of final logMAR VA, eyes with initial VA of ≥20/200 (Mann-Whitney *U* test: *P* = 0.003), focal type of endophthalmitis (Kruskal-Wallis test: *P* = 0.004), and diabetes mellitus (Mann-Whitney *U* test: *P* = 0.014) were also significantly associated with final logMAR VA. An intravitreal injection and vitrectomy were not associated with good visual outcomes (Mann-Whitney *U* test: *P* = 0.462, *P* = 0.947, resp.). There was no significant difference in the visual outcomes between gram-positive and gram-negative infections and between MRSA and MSSA infections (Mann-Whitney *U* test; *P* = 0.969, *P* = 0.758, resp.). A longer time between the onset of ocular symptoms and intravitreal antibiotic injection was weakly correlated with worse visual outcomes (Spearman: *P* = 0.056). However, the interval between the diagnosis of endophthalmitis and intravitreal injection was not significantly correlated with the final visual outcome (Spearman: *P* = 0.616). The correlations between the final visual outcome and age, interval between the onset of ocular symptoms and initial examination by an ophthalmologist and vitrectomy were not significant (Spearman: *P* = 0.275, *P* = 0.176, and *P* = 0.216, resp.). Among continuous values including age, initial logMAR VA, follow-up period, and interval between onset of ocular symptoms and intravitreal antibiotic injection, multiple regression analysis detected that initial logMAR VA was significantly associated with final logMAR VA in eyes with intravitreal antibiotic injection (*P* = 0.001, 95% CI: 0.437–1.344). The interval between onset of ocular symptoms and intravitreal antibiotic injection was weakly associated with final logMAR VA (*P* = 0.097, 95% CI: −0.006–0.064) by multiple regression analysis. In eyes without intravitreal antibiotic injection, initial logMAR VA (*P* = 0.022, 95% CI: 0.198–1.798) was detected as a related factor for final logMAR VA by multiple regression analysis.

The two eyes infected with* K. pneumoniae* were enucleated (Patients 1 and 12). One patient (Patient 13) died before discharge. This patient had adult onset Still's disease with administration of high-dose steroids, but disseminated MRSA ultimately developed.

## 4. Discussion

EBE is a rare form of infection which occurs when organism reaches the eye via the bloodstream and then crosses the blood-ocular barrier. Although the visual prognosis was reported to be very poor [1], the current study revealed that it mainly depended on initial VA.

In our 27 eyes of 21 case series, extraocular infectious foci were identified in 81.0% and infective endocarditis (14.3%) was the common extraocular infection. It has been reported that the extraocular sites of bacterial endophthalmitis were the endocardium, liver, lung, central nervous system, and the renal and urinary tracts [3]. Okada et al. and Yonekawa et al. reported that the most common site of EBE was the heart with infectious endocarditis [2, 8]. Infectious endocarditis is considered to be one of the most serious infections in the Western world, and the causative organisms were most often staphylococci, streptococci, and enterococci [16]. Kuriyan et al. reported that, in spite of prompt vancomycin treatment, most patients with ocular* Streptococcus* infections had poor VA [17]. In our study, EBE with infective endocarditis was caused by* S. equisimilis*, a subspecies of Group G* Streptococcus*,* S. agalactiae, *a subspecies of Group B* Streptococcus*, and an unidentified organism in one patient each and the visual outcomes varied from phthisis to 20/16. Despite intravitreal vancomycin injection, 1 patient with initial VA of light projection ended in phthisis.

Gram-positive organisms are more common in North American and European cases of EBE [2, 3, 18]. In the current study, we also found that gram-positive organisms were the most common bacterial pathogens, especially* S. aureus* which was isolated from 11 (MRSA in 8 and MSSA in 3) patients. Visual outcomes are generally poor in EBE caused by MRSA [19]. Ho et al. reported a high incidence of retinal detachments in eyes with endogenous MRSA endophthalmitis, and almost one-half of the affected patients eventually required enucleation or evisceration [5]. In our 9 eyes (7 patients) with MRSA in whom we could examine VA measurement, 7 eyes were treated with both systemic and intravitreal antibiotics. Eight eyes had a final VA better than 20/200, and that is better than that reported earlier [5, 19]. One eye with initial VA of light perception ended in phthisis. No retinal detachments developed and none had to be enucleated during the follow-up period. There was no significant difference in the visual outcomes between MRSA and MSSA infections in the current study.

Gram-negative microorganisms have been reported to be the main causative pathogens of endogenous endophthalmitis in East Asians [4, 20]. Among the gram-negative microorganisms, there has been an increase in* K. pneumonia* as the causative organism in endogenous endophthalmitis. Thus, it has become an important pathogen in recent years in Asian countries [1, 3, 4, 20–25], and the incidence of infections by* K. pneumonia* in eyes with endogenous endophthalmitis was 50% to 61% [4, 26]. A national clinical study in 19 hospitals on bacterial and fungal endophthalmitis during the past-5-to-20-year period (until 1988) in Japan showed that the incidence of endogenous endophthalmitis caused by* K. pneumonia* was 25% (5 of 20 cases) [27]. Torisaki et al. performed a literature search on EBE caused by* K. pneumoniae* published between 1989 and 1998 in Japan [28]. A total of 26 references (single case and small case series) on 30 patients (41 eyes) were found and the visual outcome of all except one was poor with 30 of 41 eyes having a final VA of no light perception [28]. In our study,* K. pneumoniae* was found in only 2 patients (9.5%), one with liver abscess and one with epididymitis. Initial VAs were counting finger and light perception, respectively, and both lost light perception during follow-up. It has been documented that* Klebsiella *sp. has worse prognosis [4, 21, 22]. Although initial VA in 2 patients with* K. pneumoniae* was poor in our study,* K. pneumoniae* infection itself may have worse prognosis. Recent experimental models focused on EBE caused by* K. pneumoniae *concluded that* K. pneumoniae's *ability to disrupt retinal function and generate an inflammatory response, thus causing further damage, can be attributed to its capsule. Moreover, they demonstrated that bacterial products, such as endotoxin, present on the surface of* K. pneumonia*, further stimulate the host inflammatory response [29, 30]. The reason for the high incidence of this infection in Asia compared with non-Asia has not been determined [4, 26].

The most common systemic condition associated with bacterial endophthalmitis was diabetes mellitus followed by hypertension, cardiac disease, gastrointestinal disorders, and urological diseases [2]. Jackson et al. also reported that the most common predisposing medical condition was diabetes mellitus (62%) including type II diabetes (42%) in a literature review and its presence was significantly associated with poor VA [3]. Although the most common predisposing medical condition was diabetes mellitus (61.9%) in the current study, patients with diabetes mellitus had significantly better final logMAR VA (Mann-Whitney *U* test: *P* = 0.014). Those patients also had significantly better initial logMAR VA (Mann-Whitney *U* test: *P* = 0.022) and 12 of 17 eyes with diabetes mellitus were the focal type of endophthalmitis.

In patients in whom we could examine VA, the incidence of the posterior focal type endophthalmitis was 48.0% (12 eyes) and eyes with the focal type of endophthalmitis were more likely to have a final visual acuity of ≥20/200 than those with posterior diffuse or panophthalmitis (Fisher's exact test: *P* = 0.011). Similar to our study, Greenwald et al. reported the posterior focal type of EBE might have a better visual outcome than the posterior diffuse or panophthalmitis with vitreal involvement [6]. Focal subretinal abscess may expand to panendophthalmitis, if not treated. However, our result may not necessarily represent the natural course of EBE (from focal to diffuse posterior then to panophthalmitis). Natural course of intraocular EBE expansion must be elucidated.

EBE develops when organisms from systemic or local infections disseminate through the blood and enter the intraocular spaces through the blood-ocular barrier [22]. Thus, an early detection of the infection site and treatment of the causative organism are very important [12]. Systemic antibiotics treat the distant foci of infection and prevent continued bacteremia, thereby reducing the chances of an invasion of the eye [20]. Some patients were treated with intravitreal antibiotics as well as with systemic antibiotics, while others were treated with intravitreal antibiotics and vitrectomy as the initial treatment modality in previous [1, 4, 12] and our studies. Yonekawa et al. recommended intravitreal antibiotics administered within 24 h to supplement the systemic antibiotics [8]. In our patients, the interval between the onset of ocular symptoms and the intravitreal antibiotic injection was weakly correlated with the final visual outcome (Spearman: *P* = 0.056 and multiple regression analysis: *P* = 0.097). For 15 (55.5%) of 27 eyes, intravitreal antibiotics were used. None (0%) of 9 eyes receiving intravitreal antibiotics (with systemic antibiotic, but not vitrectomy) underwent enucleation, compared with 3 (25%) of 12 eyes who had systemic treatment alone (no vitrectomy). Recent review [1] also mentioned that intravitreal antibiotics may be associated with a trend for fewer enucleation surgeries. Although these data suggested an association between intravitreal antibiotics and preservation of the eye, they cannot establish a causal link.

Romero et al. recommended surgical intervention for patients infected with especially virulent organisms, or visual acuity of ≤20/400, or severe vitreous involvement as in advanced stages, for example, posterior diffuse endophthalmitis or panophthalmitis [7]. In the current study, 5 eyes with initial VA of ≤20/400 (4 with posterior diffuse type infection and 1 with focal type infection) and 1 eye with posterior diffuse type infection caused by* Neisseria *sp. underwent vitrectomy. Final VAs varied from NLP (phthisis) to 20/20. Three eyes with initial VA of hand motion or light perception and with posterior diffuse endophthalmitis ended in final VA of less than CF. Surgical interventions for EBE are difficult because most patients are in poor general condition and are a high risk for general anesthesia [12]. In our cases, 4 of 6 eyes underwent vitrectomy more than 7 days after the onset of the ocular symptoms, and the visual acuity outcomes were poor except for one eye (Patient 18). Because of patient condition, the timing of surgery was delayed. On the other hand, Wong et al. reported that the final visual outcome was unrelated to the use of vitrectomy in the management and only the virulence of the organism predicted the outcome [20]. At present, the efficacy of immediate ocular therapies including vitrectomy and intravitreal antibiotics against EBE is still controversial [22].

The most common systemic finding was fever including cold-like symptoms (76.2% in our study and 57.7% in the systemic review) [1]. At the first visit to a doctor, a high body temperature (>38°C) (76.2%), elevated C-reactive protein (76.2%), and a high white blood cell count (52.3%) were observed in the current study. Diabetes mellitus was the most common medical condition in our study (61.9%) and the systemic review (33%) [1]. A funduscopic examination should be performed as soon as possible after the onset of symptoms in those with elevated CRP, high WBC, unknown origin of fever, and/or a history of diabetic mellitus. The most common ocular symptoms were decreased vision (66.7%), floaters (23.8%), and pain (9.5%) in our study and those are similar to others [1].

In the current study, a final VA of 20/200 or better was achieved in 64% and the median final VA was 20/40. Visual outcomes seems to be better than a recent review [1] of 342 cases of EBE (initial VAs were not mentioned), in which the median final VA was 20/100, with 44% worse than 20/200. The reasons for better visual outcome in our study might be as follows. There were only two eyes with* Klebsiella *sp. which was reported to be worse prognosis [4, 21, 22]. In Japan, physicians routinely send patients to an ophthalmologist for funduscopic examination when patients have diabetes mellitus. Patients were also referred to an ophthalmologist from physicians according to the Guidelines for Management of Deep-Seated Mycoses 2007 in Japan, when endogenous endophthalmitis was suspected. As a result, most patients were referred to an ophthalmologist earlier and early systemic and/or intravitreal antibiotics could be instituted. The number of eyes with initial VA of ≥20/200 was 10 (40.0%) and final VA of ≥20/200 was 16 (64.0%) and 20/40 or better was 11 (44.4%) of 25 eyes in our study. In Wu's study [4], the number of eyes with initial VA of ≥20/200 was 0 (0%) of 15 eyes with EBE and final VA of ≥20/200 was 3 (20.0%). Moreover our statistical analysis clearly showed initial VA as a related factor for visual outcome. However, it has been reported that early diagnosis alone usually did not result in good visual outcome [2]. Other than a delay in diagnosis and treatment, factors associated with the poor prognosis may include the virulence of the causative organisms and compromised host conditions.

The limitations of this study are those inherent in retrospective studies, including lack of controls and uniform protocol. However large randomized control trial for this rare disease is not practical; observational case series will be an important source for developing future treatment guidelines.

## 5. Conclusions

EBE is a rare but often devastating ocular and systemic disorder. In contrast with previous studies, our study showed better visual outcome. The prognosis may depend on initial VA, extent of ocular involvement, and an interval between the onset of ocular symptoms and intravitreal antibiotic injection. Thus, early diagnosis and early intravitreal injection of antibiotics supplement to immediate systemic antibiotics might lead to a favorable visual outcome. A higher index of suspicion should be maintained by clinicians. All patients with bacteremia, including suspected cases, should have at least one dilated retinal examination early in the course of therapy preferably performed by an ophthalmologist.

## Figures and Tables

**Table 1:** Patient characteristics and systemic features.

Number	Age (years)	Sex	Underlying medical condition	Presumed infection source	CRP (mg/dL)	WBC (/*μ*L)	*β*-D-glucan (pg/mL)	Systemic steroid	Days from onset of symptoms to initial examination (ophthalmologist)
1	72	M	HT, cardiac disease	Liver abscess	8.18	13100	ND	−	4 (same day)
2	60	F	DM	Psoas abscess	21.42	15890	ND	−	0 (6)
3	72	F	DM, uterine cancer	Peritonitis	6.8	18100	ND	−	3 (same day)
4^†^	76	M	DM	Pneumonia	30.23	14230	<5.0	−	29 (same day)
5^†^	80	M	Gastric cancer	Catheter-related	9.98	12700	ND	−	6 (same day)
6	73	M	Autoimmune hemolytic anemia, DM, HT	Unknown (many organs)	0.34	12630	230	+	2 (2)^‡^
7	74	F	Cardiac disease	Infective endocarditis	10.65	11850	<5.0	−	5 (14)
8^†^	77	M	Colon cancer, intestinal perforation	Peritonitis	2.69	9390	6.3	+	28 (same day)
9	60	M	DM, HT, hemodialysis, gastric ulcer, infectious spondylitis	Soft tissue infection	7.68	4490	15.4	−	6 (same day)
10	70	F	Lung disease, HT	Pneumonia	10.93	14200	6.6	−	2 (2)^‡^
11	58	M	DM, cardiac disease	Infective endocarditis	18.71	12050	5.3	−	1 (8)
12	73	M	Urological disease	Urinary tract infection	16.15	19320	ND	−	4 (same day)
13^†^	41	F	Adult onset Still's disease	Unknown	0.8	24560	<5.0	+	2 (30)
14^†^	59	M	DM, hemodialysis	Burn	9.32	9400	ND	−	13 (same day)
15	71	F	DM, HT, cardiac disease, rheumatoid arthritis	Infective endocarditis	18.96	15500	ND	−	1 (same day)
16	79	M	DM, colon cancer	Unknown	10.09	8230	<5.0	−	1 (1)^‡^
17^†^	71	M	DM, HT, renal failure, liver cirrhosis, rupture of umbilical hernia	Catheter-related	12.25	8470	9.1	−	6 (7)
18	78	M	Renal failure	Unknown	0.62	7300	11.9	+	5 (same day)
19^†^	55	M	DM, HT	Head trauma	14.49	23210	<5.0	−	2 (2)^‡^
20	68	F	DM	Leg abscess	0.09	11610	<5.0	+	3 (3)^‡^
21^†^	72	M	DM, cardiac disease	Renal trauma	3.69	9770	19.6	−	2 (same day)

^†^Hospital-acquired patient; CRP, C-reactive protein; WBC, white blood cell; M, male; F, female; DM, diabetes mellitus; HT, hypertension; ND, not done; ^‡^initially consulted an ophthalmologist.

**Table 2:** Ocular features, microbiological findings, and treatments.

Number	Eye	Type of EBE^*※*1^	Organism	Culture			Antibiotics		Surgery^*※*3^	Initial VA	Outcome (follow-up in weeks)
				Blood	Vitreous	Other positive cultures	Systemic	Intravitreal^*※*2^			
1	OD	Pan	*K. pneumoniae *	−	+		ASPC	None	Enucleation (7)	CF	NLP (17)
2	OS	PD	*S.aureus *	+	ND	Psoas (*S.aureus*)	PAPM/PM, VCM, CLDM, CTRX	CAZ (<1)	—	20/25	20/16 (520)
3	OD	PF (N-L-T)	*S.agalactiae, *CNS	+	+		IPM/CS, ITCZ	VCM + CAZ^#^ (1)	Vit + PEA (1)	20/600	20/20 (35)
4^†^	OS	PD	MRSA	+	− (AQ−)	Urine (MRSA)	PAPM/BP, VCM	VCM + CAZ^#^ (1)	Vit + PEA + s/o (1)	CF	20/200 (3)
5^†^	OS	PD	*S.aureus, S.agalactiae *	+	ND		CEZ, ST, MEPM, F-FLCZ, CDTR-PI	None	—	20/600	20/25 (78)
6	OS	PF (Pos)	*Nocardia farcinica *	+	−^*※*4^		MCFG, F-FLCZ, MEPM, IPM/CS, ST	None	—	20/50	20/40 (313)^§1^
7	OS	Pan	*S.equisimilis *	+	+ (AQ+)		PCG, CTRX, GM, TAZ/PIPC	VCM + MEPM (<1)	—	LP	Phthisis (57)
8^†^	OD	PD	MRSA	ND	−	IV cannula (MRSA)	CPR, VCM	VCM + CAZ^#^ (9)	Vit + PEA (37)	LP	Phthisis (2)
9	OD	PF (Pos)	MRSA	+	ND		CAZ, BIPM, CEZ, CTM	None	—	20/20	20/25 (35)
10	OD OS	PD PD	*S.pneumoniae *	+	− −		CPR, TAZ/PIPC, PIPC, FMOX, CFPN-PI	VCM + CAZ^#^ (28) VCM + CAZ^#^ (8 & 15)	Vit + PEA + s/o (30) Vit + PEA (10)	LP HM	LP (196) HM (196)
11	OD OS	PF PF	Unknown	−	ND	Sputum (MRSA)	FOM, CZOP, CTRX, GM, VCM, LZD	None None	—	20/32 20/28	20/16 (113) 20/16 (113)
12	OS	Pan	*K. pneumoniae *	−	+ (AQ+)		CPR, CEZ, IPM/CS. CTRX	None	Enucleation (9)	LP	NLP (4)
13^†^	OD OS	PF (T) PD	MRSA	+	ND		CFDN, MEPM, MINO, MCFG, F-FLCZ, BIPM, LVFX, TAZ/PIPC, VCM, LZD	None None	—	ND	Death (3)
14^†^	OD OS	PF (N) PF (N)	MRSA	+	(AQ−) (AQ−)		VCM, TEIC	VCM + CAZ (8 & 15) VCM + CAZ (15)	—	20/500 20/500	20/250 (14) 20/200 (14)
15	OS	PF (N)	*S.agalactiae *	+	ND		AZM, IPM/CS, GM	None	—	CF	20/20 (33)
16	OD	PD	Unknown	−	(AQ−)		CZOP	VCM + CAZ (4)	—	HM	HM (15)
17^†^	OS	PD	MRSA	+	(AQ−)		VCM, F-FLCZ	VCM (<1 & 2)	—	HM	20/60 (7)
18	OS	PD	*Neisseria *sp.	+ ^‡^ *S.aureus *	+		VCM, MEPM, CEZ, MCFG, FLCZ	VCM + CAZ^#^ (22)	Vit (22)	20/25	20/20 (7)
19^†^	OD OS	PF (SupN) Pan	*S.aureus *	+	+		MINO, DRPM, LZD, CEZ	VCM + CAZ (<1) None	— Enucleation (23)	20/20 NLP	20/100^§2^ (67) NLP (67)
20	OS	PF (SupN)	MRSA	−	ND	Leg abscess (MRSA)	ST, CTRX, LZD	None	—	20/20	20/20 (44)
21^†^	OD OS	PF (T) PF (T)	MRSA, *E.cloacae *	+	ND	IV cannula (MRSA)	CFPM, F-FLCZ, LZD, DAP, RFP, ST	VCM (9) VCM (16)	—	20/100 20/40	20/20 (39) 20/50^§3^ (39)

^†^Hospital-acquired patient, ^*※*1^site of subretinal abscess in parentheses, ^*※*2^number in parentheses represents days from diagnosis of endophthalmitis, ^*※*3^number in parentheses represents days from onset of ocular symptoms, and ^*※*4^diagnostic vitrectomy; OD, right eye; OS, left eye; EBE, endogenous bacterial endophthalmitis; Pan, panophthalmitis; PD, posterior diffuse; PF, posterior focal: N, nasal; T, temporal; L, lower; Pos, posterior; SupT, superior temporal; SupN, superior nasal; CNS, coagulase-negative *Staphylococcus*; MRSA, methicillin-resistant *Staphylococcus aureus*; AQ, aqueous; ND, not done; IV, intravenous; VA, visual acuity; Vit, vitrectomy; PEA, phacoemulsification and aspiration; s/o, silicone oil; NLP, no light perception; CF, counting fingers; LP, light perception; HM, hand motion; ^§1^cataract and epiretinal membrane; ^§2^vitreous hemorrhage; ^§3^cataract; ^#^vitrectomy and intravitreal injection; ^‡^not detected *Neisseria *sp. from blood culture; TAZ/PIPC, tazobactam/piperacillin; PIPC, piperacillin; PCG, benzyl penicillin; ASPC, aspoxicillin; CTRX, ceftriaxone; CEZ, cefazolin; CPR, cefpirome; CZOP, cefozopran; CDTR-PI, cefditoren pivoxil; CTM, cefotiam; CFDN, cefdinir; CAZ, ceftazidime; CFPM, cefepime; FMOX, flomoxef; CFPN-PI, cefcapene pivoxil; IPM/CS, imipenem/cilastatin; MEPM, meropenem; BIPM, biapenem; PAPM/BP, panipenem/betamipron; DRPM, doripenem; GM, gentamicin; AZM, azithromycin; CLDM, clindamycin; MINO, minocycline; LZD, linezolid; VCM, vancomycin; TEIC, teicoplanin; DAP, daptomycin; LVFX, levofloxacin; RFP, rifampicin; ST, sulfamethoxazole; FOM, fosfomycin; F-FLCZ, fosfluconazole; MCFG, micafungin.

**Table 3:** Isolated organism from blood, intraocular, or other cultures.

Organisms (number of cases)	Cultures							Subtotal
	Blood (*n* = 8)	Blood & vitreous (*n* = 3)	Blood, vitreous, aqueous & others (*n* = 1)	Blood & others (*n* = 5)	Vitreous (*n* = 2)	Vitreous & aqueous (*n* = 1)	Others (*n* = 2)	
Gram-positive (*n* = 18)								
* Staphylococcus aureus *(MRSA)	3			3			2	8
* Staphylococcus aureus *(MSSA)	1	1		1				3
CSN		1						1
* Streptococcus agalactiae *	2	1						3
* Streptococcus pneumoniae *	1							1
* Streptococcus equisimilis *			1					1
* Nocardia farcinica *	1							1

Gram-negative (*n* = 4)								
* Neisseria *sp.					1			1
* Klebsiella pneumoniae *					1	1		2
* Enterobacter cloacae *				1				1

CNS, coagulase-negative *Staphylococcus*; MRSA, methicillin-resistant *Staphylococcus  aureus*; MSSA, methicillin-sensitive *S. aureus.*

**Table 4:** Prognostic factors associated with good visual outcomes.

Factors	Final visual outcome		Odds ratio (95% CI)	*P*
	≥20/200	<20/200		
Sex				
Male	12	6	1.50 (0.25–8.98)	1.00
Female	4	3		
Eye				
Right	5	5	0.36 (0.07–1.97)	0.397
Left	11	4		
Initial visual acuity				
≥20/200	10	0	—	0.003
<20/200	6	9		
Extent of ocular involvement				
Panophthalmitis	0	4	—	0.003^#^
Posterior focal	5	4		
Posterior diffuse	11	1		
Diabetic mellitus				
Yes	14	3	14.00 (1.84–106.47)	0.010
No	2	6		
Hypertension				
Yes	5	2	1.59 (0.24–10.57)	1.00
No	11	7		
Systemic steroid treatment				
Yes	3	1	1.85 (0.16–20.94)	1.00
No	13	8		
Infection place				
Community-acquired endophthalmitis	7	6	0.39 (0.08–2.01)	0.411
Hospital-acquired endophthalmitis	9	3		
Causative organisms				
Gram stain positive	11	6	1.44 (0.19–11.04)	1.00
Gram stain negative	3	2		
*K. pneumonia *				
Yes	0	2	0.0 (0.0–1.00)	0.121
No (others)	14	6		
Intravitreal antibiotics				
Yes	9	6	0.64 (0.13–43.32)	0.691
No	7	3		
Interval between diagnosis and intravitreal				
antibiotics				
≤1 day	5	1	6.25 (0.50–77.50)	0.287
>1 day	4	5		
Vitrectomy				
Yes	3	3	0.46 (0.08–2.66)	0.630
No	13	6		

Fisher's exact test.

^#^Chi-square test.
